# Association between hypothyroidism and obstructive sleep apnea: a bidirectional Mendelian randomization study combined with the geo database

**DOI:** 10.3389/fneur.2024.1420391

**Published:** 2024-12-10

**Authors:** Mingyu Zhao, Xu Huang, Hu Zheng, Yuhang Cai, Wenjia Han, Yuanyin Wang, Ran Chen

**Affiliations:** Key Laboratory of Oral Diseases Research of Anhui Province, College & Hospital of Stomatology, Anhui Medical University, Hefei, China

**Keywords:** hypothyroidism, obstructive sleep apnea, GEO, Mendelian randomization, causality

## Abstract

**Background:**

The causal relationship between hypothyroidism and obstructive sleep apnea (OSA) remains controversial. Therefore, our research used a bidirectional Mendelian randomization (MR) method in an attempt to determine the causal relationship between hypothyroidism and OSA.

**Methods:**

From the publicly accessible genome-wide association analysis (GWAS) summary database, we obtained single nucleotide polymorphism (SNPs) data pertaining to hypothyroidism and OSA. Inverse variance weighting (IVW) was the principal method of analysis utilized, with validation also conducted via weighted median, MR-Egger, simple model, and weighted model approaches. To further evaluate the robustness of the results, heterogeneity testing, pleiotropy testing, and the “leave-one-out” sensitivity analysis were performed. Differentially expressed genes (DEGs) from the OSA dataset (GSE135917) and hypothyroidism dataset (GSE176153) derived from the Gene Expression Omnibus (GEO) database were screened using the “limma” package. The “clusterProfiler” and “GO plot” packages were used for further enrichment analysis in order to validate the findings of the MR study. The Cytoscape software was utilized to build a protein–protein interaction (PPI) network of DEGs and to screen for hub genes.

**Results:**

The MR analysis showed that genetically predicted hypothyroidism was associated with an increased risk of OSA [IVW odds ratio (OR) = 1.734; 95% confidence interval (CI) = 1.073–2.801; *p* = 0.025]. The trend of the outcomes of the other approaches is consistent with the trend of the IVW outcome. However, the reverse MR analysis suggested no evidence for the causal effect of OSA on hypothyroidism (IVW OR = 1.002, 95% CI: 0.996–1.009, *p* = 0.454). The robustness of the results was confirmed by the sensitivity analysis. Bioinformatics analysis revealed that there were DEGs that hypothyroidism and OSA have in common.

**Conclusion:**

Our findings suggested that hypothyroidism may increase the risk of OSA, while the effect of OSA on hypothyroidism was not found in this MR study. Thus, patients with hypothyroidism should be enhanced with screening for OSA for early diagnosis and appropriate treatment.

## Introduction

Obstructive sleep apnea (OSA) is a widely observed disease that is distinguished by repeated, partial, or total blockage of the upper respiratory tract while sleeping. This obstruction causes intermittent decreases in blood oxygen saturation, blood oxygen partial pressure, as well as hypercapnia (1). About 1 billion individuals between aged of 30 and 69 are estimated to have obstructive sleep apnea (2). Individuals suffering OSA are more probable to experience daytime sleepiness, lethargy, poor concentration, memory loss, or headaches, all of which can negatively impact quality of life and life expectancy (3). In addition, OSA has the potential to result in complications such as stroke, type 2 diabetes mellitus, hypertension, and even nocturnal abrupt death (4–6).

It has been shown that OSA is associated with thyroid disease, especially hypothyroidism, and that patients with hypothyroidism and those with OSA often exhibit similar symptoms (7). Hypothyroidism is a systemic hypometabolic syndrome resulting from thyroid hormoneemia or thyroid hormone resistance due to autoimmunity, iodine metabolism disorders, thyroid surgery, etc. (8). Clinical hypothyroidism is a prevalent condition, with prevalence rates ranging from 0.3 to 3.7% in the overall population of United States and from 0.2 to 5.3% in the Europe (9). Hypothyroidism can result in a higher risk to hyperlipidemia, cardiovascular illness, reproductive abnormalities, somatic and neuromuscular symptoms and other unfavorable consequences (10).

Although the association between hypothyroidism and OSA has been studied, it remains unclear, and a recent meta-analysis also suggests that the relationship between OSA and thyroid dysfunction remains controversial (11). Some observational studies have demonstrated a greater occurrence of hypothyroidism in individuals with OSA, while hypothyroidism can also increase the risk to have OSA (12–14). A multivariate logistic regression analysis by Thavaraputta et al. (2019) demonstrated a strong correlation between OSA and hypothyroidism after adjusting for potential confounding variables (15). However, a study evaluating 271 patients with OSA by Bahammam et al. revealed that the incidence of clinical hypothyroidism newly diagnosed in patients with OSA was comparatively low (16). Besides, the present studies are predominantly grounded in observational epidemiology methodologies. Limitations of traditional observational research methods include susceptibility to reverse causality and unmeasured confounders. Because of the potential bias of confounders, the correlation inference of these previous observational studies may be limited, and the conclusions may even be considered controversial (17). Thus, further research is still needed to determine the causal relationship between hypothyroidism and OSA.

Mendelian randomization is an effective analytical method that utilizes genetic variations as instrumental variables (IVs) (18). Because genetic mutations are innate and independent of environmental factors, MR research approach could effectively manage confounding factor interference, similarly to randomized trials. Moreover, since genetic mutations can influence outcomes, but outcomes are unable to influence genes, no inference about reverse causality could be derived. In order to ensure the validity of the causal relationships obtained from MR studies, the instrumental variables (IVs) must meet three fundamental assumptions: (1) The relevance assumption states that IVs should be strongly correlated with the exposure traits; (2) The independence assumption states that IVs should not be correlated with confounding variables.; and (3) The exclusivity assumption states that IVs should only affect outcomes via exposure variables and not via other pathways (19). The identification of thousands of genetic variations associated with a variety of complicated diseases through genome-wide association studies (GWASs) has boosted the use of MR (20). In this study, we investigated the causal relationship between hypothyroidism and OSA by employing a bidirectional MR analysis with large-scale GWAS data and verifying the results of the MR analysis with bioinformatics analysis.

## Materials and methods

### GWAS data acquisition

The hypothyroidism GWAS data (GWAS ID: ukb-b-19732) was acquired from the IEU GWAS database.^1^ There were 462,933 individuals of European descent in the hypothyroidism GWAS data, comprising 22,687 cases and 440,0.246 controls. The definition of hypothyroidism cases was established using clinical diagnosis and self-report measures. The OSA GWAS data were acquired from Round 9 of the FinnGen consortium and included 38,998 OSA patients and 336,659 controls from Europe. The complete OSA GWAS was made available to the public through the FinnGen research project.^2^ The project’s main objective is to identify new targets for treatment by analyzing genotype–phenotype correlations. The OSA cases was diagnosed according to International Statistical Classification of Diseases (ICD) codes (ICD-9: 3472 A, ICD-10: G47.3). More specifically, OSA cases were identified using clinical examination, subjective symptoms, and sleep registry with a respiratory event index (REI) or apnea-hypopnea index (AHI) of no less than 5 per hour. Table 1 presents the details of the two GWAS datasets. As a reanalysis of previously gathered and published data, this research did not need further ethical clearance.

**Table 1:** Details on GWAS datasets that were used for the Mendelian randomization analysis.

Trait	Consortium	Case/Control	Sample size	Population	Year
Hypothyroidism	MRC-IEU	22,687/440,246	462,933	European	2018
OSA	FinnGen	38,998/336,659	375,657	European	2023

GWAS, genome-wide association study; OSA, obstructive sleep apnea.

### Selection of instrumental variables

Rigorous SNP screening was performed for the forward analysis with hypothyroidism as the exposure. Firstly, we selected independent SNPs that showed a significant correlation with the exposure variable, meeting the threshold of genome-wide significance [*p* < 5 × 10–8, linkage disequilibrium (LD) r2 < 0.001 within a 10,000 kb window]. Secondly, the PhenoScanner V2 database^3^ was used to identify and exclude SNPs that may be associated with confounding. Thirdly, in order to determine whether or not the retained SNPs were susceptible to weak instrument bias, the F statistic was calculated and utilized as in the prior research (21). SNPs with a F statistic below 10 were deemed to be weak instruments and were subsequently excluded from the analysis (22). Fourthly, the retained SNPs were extracted from the outcome dataset, with undetected SNPs and those directly associated with the outcome variable (*p* < 5 × 10–8) being excluded. Fifthly, to exclude those that were incompatible or palindromic, we harmonized the SNPs. Mendelian randomization analysis was finally conducted on the SNPs that remained after the previously mentioned filtration stages. The instrument selection for reverse analysis was identical to that of the forward analysis.

### MR analysis

In both forward and reverse MR analyses, the inverse variance weighted (IVW) method was employed as the principal analysis to explore the causal relationship between hypothyroidism and OSA. The IVW approach has the greatest statistical power if all assumptions are satisfied (23). To assess the robustness of the MR estimates, several of other well-established MR approaches, include MR-Egger, weighted median, simple model, and weighted model, were used in comparison with the IVW method’s results. Assuming instrument strength independent of direct influence, the MR-Egger approach can consistently provide results (24). Even though 50% of IVs are invalid, the weighted median approach can still result in a consistent estimate (25). Consistency of direction and magnitude of several kinds of methods can strengthen the robustness of inference of causality.

In order to assess whether the causality obtained was biased, we further performed sensitivity analyses. The Cochran’s *Q* test was employed to assess heterogeneity. When heterogeneity was identified, the random-effect IVW approach was used. The MR-Egger regression test was subsequently employed to evaluate horizontal pleiotropy. The intercept reflects pleiotropic effects across genetic variations (24). In addition, the leave-one-out analysis was conducted to find any outliers which could significantly skew the pooled IVW estimations. The “TwoSampleMR” package (version 0.5.7) in the R (version 4.3.1) were used for all MR analyses (26).

### Bioinformatic analysis

From the Gene Expression Omnibus (GEO) database,^4^ the hypothyroidism-related dataset GSE176153 and OSA-related dataset GSE135917 were extracted. The “limma” package (27) was applied to conduct differential expression analysis in order to identify Differential Expression Genes (DEGs) meeting the following criteria: *p* value <0.05 and |log2(FC)| > 0.5, and the “ggplot2” package (28) and “pheatmap” package (29) were employed to visualize the results. The results of MR analyses were verified by using a Venn diagram to determine whether common DEGs existed between hypothyroidism-DEGs and OSA-DEGs. Gene Ontology (GO) enrichment analysis was performed on the derived common DEGs through the “clusterProfiler” package (30) and “GO plot” package (31) in order to identify the principal pathways and functions implicated in the common DEGs. All of the packages used above are part of the R software statistical environment. The STRING database,^5^ a free online tool for identifying and predicting protein interactions, was used to build the PPI network of the shared DEGs. Cytoscape software (version 3.9.0) was employed to display the network, and the hub genes were identified using the Cytohubba plugin in Cytoscape.

## Results

### Selection of genetic instrument

According to the predetermined criteria, forward MR analysis was performed on 110 SNPs linked to hypothyroidism, while reverse MR analysis was performed on 18 SNPs linked to OSA. All of the F statistics were more than 10, indicating that our research did not include any weak instruments. Supplementary Tables S1, S2 provide details on the included instrumentation variations on hypothyroidism and OSA.

### Casual effect of hypothyroidism on OSA

**Figure 1:**
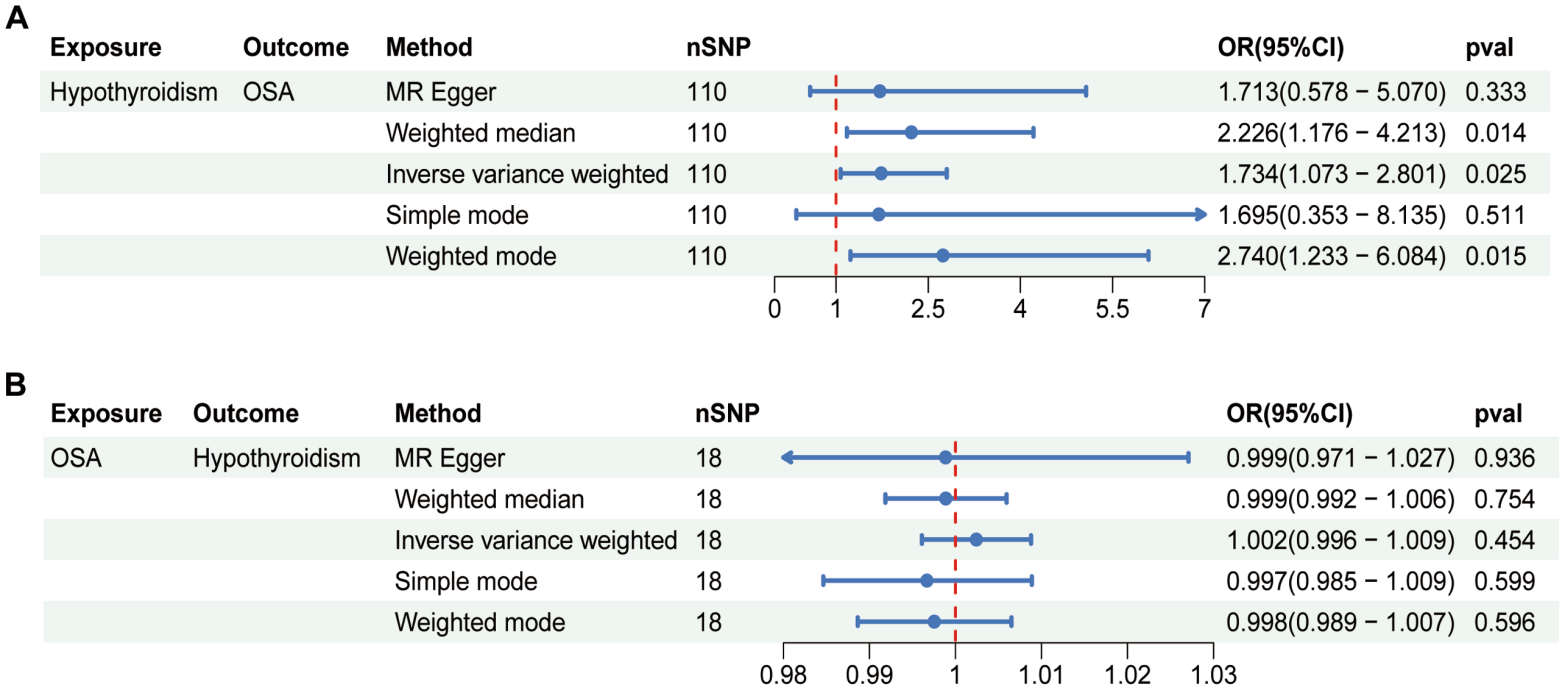
Bidirectional causal relationship between hypothyroidism and OSA. **(A)** The causal effect of hypothyroidism on OSA. **(B)** The causal effect of OSA on hypothyroidism. OSA, obstructive sleep apnea; nSNP, number of SNPs employed in this study; OR, odds ratio; CI, confidence interval.

The MR results of hypothyroidism on OSA are listed in Figure 1A. The included IVs showed no indication of horizontal pleiotropy, according to MR-Egger regression analysis (intercept = 6.123e-05, *p* = 0.980; Table 2). The heterogeneity of the results was assessed using the IVW (Cochran’s *Q* = 174.344; *p* < 0.05) and MR-Egger (Cochran’s *Q* = 174.343; *p* < 0.05) approaches, which indicated that there was heterogeneity in the included IVs (Table 2). Therefore, the inverse variance weighted method under random effect was used to evaluate the causal associations of hypothyroidism on OSA. Genetically predicted hypothyroidism was associated with an increased risk of OSA, according to the IVW method [odds ratio (OR) = 1.734; 95% confidence interval (CI) = 1.073–2.801; *p* = 0.025]. All five approaches exhibit positive ORs and similarly directional correlations were detected using the other approaches (weighted mode, OR = 2.740, 95% CI = 1.233–6.084, *p* = 0.015; weighted median, OR = 2.226, 95% CI = 1.176–4.213, *p* = 0.014), despite the fact that the results of the simple mode and MR Egger methods were not statistically significant (MR Egger, OR = 1.713, 95% CI = 0.578–5.070, *p* = 0.333; Simple mode, OR = 1.695, 95% CI = 0.353–8.135, *p* = 0.511).

**Table 2:** Sensitivity analysis of the associations between hypothyroidism and OSA.

Exposure	Outcome	Heterogeneity test				Pleiotropy test	
		IVW		MR-Egger		MR-Egger intercept	
		*Q*	*p*-value	*Q*	*p*-value	Intercept	*p*-value
Hypothyroidism	OSA	174.344	7.099E-05	174.343	5.502E-05	6.123E-05	0.980
OSA	Hypothyroidism	33.384	0.010	33.246	0.007	2.113E-04	0.800

OSA, obstructive sleep apnea; IVW, inverse variance weighted.

**Figure 2:**
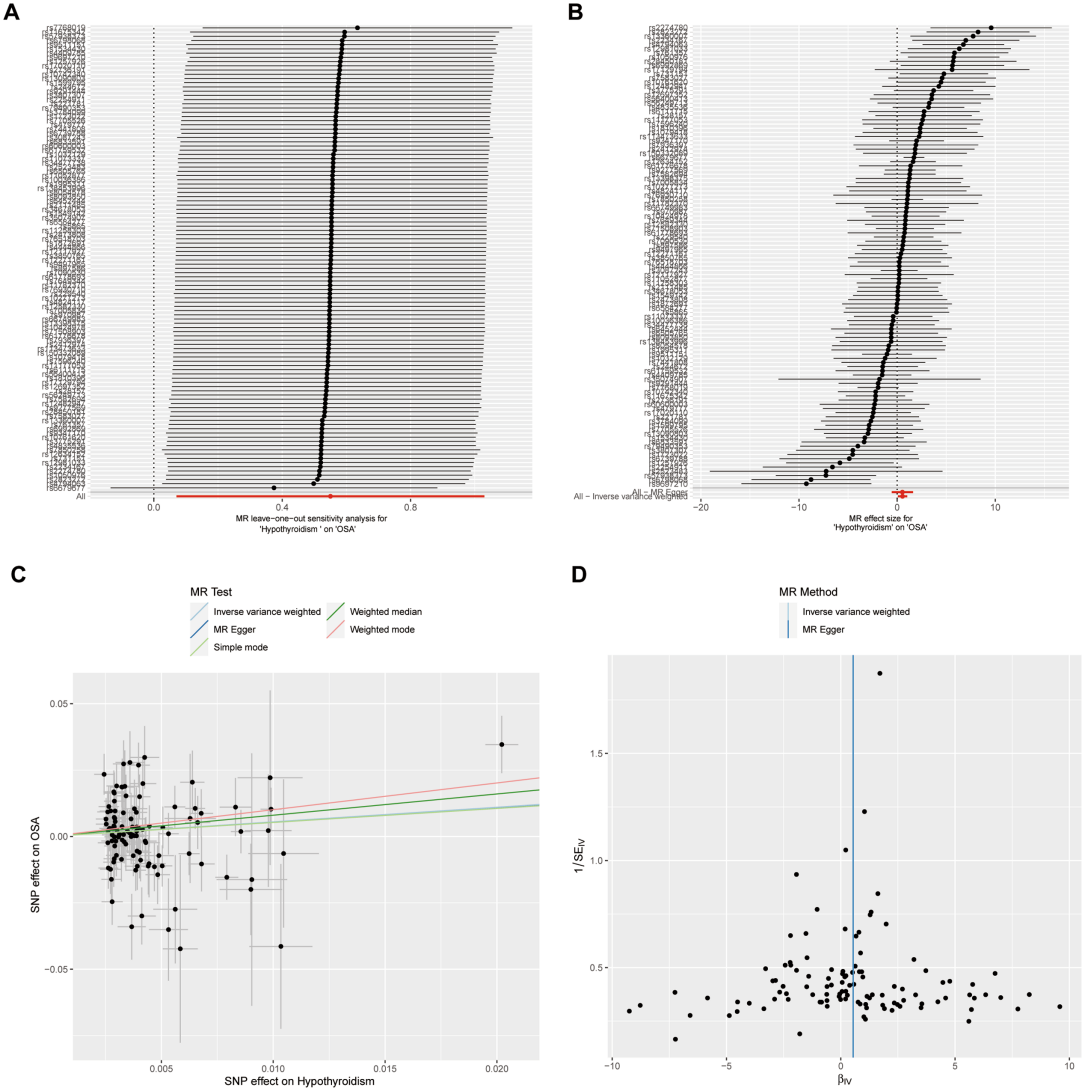
Sensitivity analysis of casual effect of hypothyroidism on OSA. **(A)** MR leave-one out analysis of hypothyroidism on OSA. **(B)** MR effect size for hypothyroidism on OSA. **(C)** MR test scatterplot of five methods. **(D)** Funnel plot of individual SNP analyses.

A leave-one-out sensitivity analysis was carried out for each SNP on the causal effect of hypothyroidism on OSA. Regardless of whether any SNP was eliminated, we observed that the outcome was always on one side of the zero line (Figure 2A). Also, by visualizing the effect size of IVW and MR-Egger using 110 SNPs, we found that the two outcomes of roughly the same (Figure 2B). In addition, the tendencies of various MR methods were comparatively consistent (Figure 2C). Finally, funnel plot demonstrated that, in both IVW and MR-Egger analysis, the distribution of each SNPs is uniformly distributed on both sides of the vertical line (Figure 2D).

### Casual effect of OSA on hypothyroidism

Reverse analysis was performed to further assess the causal relationship between OSA and hypothyroidism. The random effect IVW methods suggested no evidence for the causal effect of OSA on hypothyroidism (OR = 1.002, 95% CI = 0.996–1.009, *p* = 0.454; Figure 1B). Meanwhile, no statistical significance was observed in the results from other MR approaches (Figure 1B). According to the results of Cochran’s Q test, heterogeneity was found in sensitivity analysis (MR Egger, Q = 33.246, *p* = 0.007; IVW, Q = 33.384, *p* = 0.010; Table 2). There was no indication of horizontal pleiotropy according to the MR-Egger regression (intercept = 2.113e-04, *p* = 0.800; Table 2). The leave-one-out analysis indicated that the total effect of OSA on hypothyroidism was not altered by any one SNP. Supplementary Figure S1 displayed the leave-one-out analysis, forest, scatter, and funnel plots of the sensitivity analysis of casual effect of OSA on hypothyroidism.

### Bioinformatic analysis

**Figure 3:**
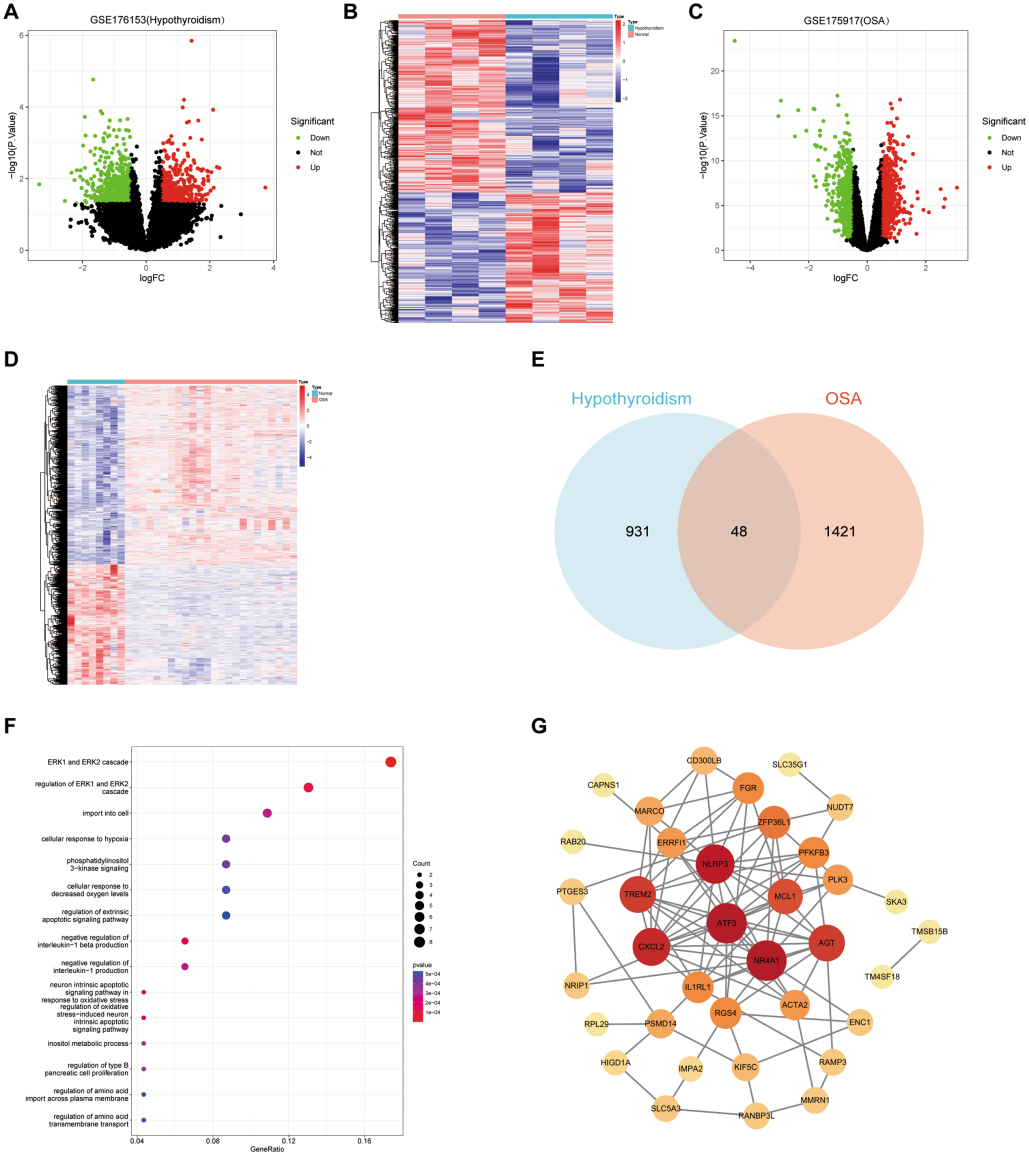
Bioinfonnatic analysis. **(A,B)** Volcano plot and heatmap of DEGs for hypothyroidism. **(C,D)** Volcano plot and heatmap of DEGs for hypothyroidism. **(E)** Venn graphic of DEGs that hypothyroidism and OSA have in common. **(F)** Gene Ontology enrichment analysis of common DEGs. **(G)** The PPI network of common DEGs. Node size increases in proportion to the number of other nodes to which it is connected.

Analysis of the hypothyroidism-related dataset GSE176153 resulted in a total of 979 DEGs, 557 of which were downregulated and 422 of which were upregulated (Figures 3A,B). Analysis of the OSA-related dataset GSE135917 resulted in a grand total of 1,469 DEGs, 587 of which were downregulated and 882 of which were elevated (Figures 3C,D). The Venn graphic shows the DEGs common to hypothyroidism and OSA (Figure 3E), including AGT, RANBP3L, ACTA2, TCEANC2, FGR, RAMP3, IMPA2, RGS4, PLK3, UCN3, PFKFB3, MARCO, CXCL2, NLRP3, TREM2, TMSB15B, SKA3, OR51E1, ZNF252P, MSTO1, IL1RL1, FAIM, RAB20, EPPIN, CAPNS1, PIGF, CD300LB, NR4A1, NRIP1, PCDHB10, ATF3, NUDT7, MOGS, ZFP36L1, PSMD14, ERRFI1, ENC1, SLC5A3, SLC35G1, HIGD1A, TM4SF18, PTGES3, OR56A4, KIF5C, MMRN1, PLBD2, MCL1, and RPL29. These findings genetically support the close association between hypothyroidism and OSA and that hypothyroidism may cause OSA. The GO enrichment analysis showed the DEGs shared between hypothyroidism and OSA primarily play roles in functions including ERK1 and ERK2 cascade, cellular response to decreased oxygen levels, cellular response to hypoxia, inositol metabolic process, negative regulation of interleukin-1 production, neuron intrinsic apoptotic signaling pathway in response to oxidative stress, and so on (Figure 3F). We employed the STRING database for PPI network analysis of common DEGs and Cytoscape software to visualize the PPI network (Figure 3G). The hub genes might potentially serve critical physiological regulatory functions in the PPI analysis. The hub genes were identified using Cytohubba plugin in Cytoscape, which determined the top 10 genes with the greatest number of connections (NR4A1, ATF3, NLRP3, CXCL2, TREM2, AGT, MCL1, ZFP36L1, IL1RL1, and PFKFB3).

## Discussion

The causal relationship between hypothyroidism and OSA is still controversial. The present study employed MR analysis to investigate the causal relationship between hypothyroidism and OSA, and the findings of the MR analysis were subsequently validated through bioinformatics analysis. Due to the influence of ethical considerations and confounding variables, conventional observational epidemiologic approaches are unable to determine accurate causal relationships (32). Therefore, the usage of MR analysis was able to compensate for the incapacity of conventional statistical approaches to eliminate confounding variables (33), and the findings of the present study were further strengthened in terms of reliability by the bioinformatics analysis validation.

In this study, the two-sample MR analysis revealed a correlation between genetically predicted hypothyroidism and an increased risk of OSA. This aligns with the results reported in several prior research investigations (34, 35). Clinical researchers have identified a number of clinical characteristics shared by hypothyroidism and OSA, including obesity, apathy, diminished cognitive function, and excessive daytime lethargy (12). These symptoms in hypothyroidism are due to a drop in basal metabolic rate and a reduction in sympathetic nervous system activity. Regarding OSA, these symptoms are the result of repetitive nocturnal episodes of cessation of breathing, which cause increased carbon dioxide levels, intermittent hypoxia, disturbances in sleep structure, and frequent awakenings (36, 37). The effect of thyroid hormones on the proper functioning of the central nervous system is very significant. Researchers have found that patients with Hashimoto’s encephalopathy, which is expected to be the most serious neurological complication in autoimmune hypothyroidism, present with electroencephalography abnormalities and disturbances in the metabolic composition of the brain: a notable decrease in the Nacetylaspartate/creatine ratio in the white matter of the left parietal lobe and the posterior cingulate regions (38). This may affect proper brain bioelectrical function and sleep. In addition, several presumptions from previous studies may explain the increased risk of OSA caused by hypothyroidism. First, hypothyroidism can cause muscle dysfunction that affects respiratory muscle strength, resulting in hypotonia of the respiratory muscles (39, 40). Second, Submucosal glycosaminoglycan deposition due to hypothyroidism leads to narrowing of the pharynx (41). Furthermore, low metabolic rate due to hypothyroidism can cause obesity, and further accumulation of fat in the abdomen and neck leads to obesity hyperventilation (42). Therefore, patients with hypothyroidism should be screened for OSA for early diagnosis.

The susceptibility to hypothyroidism was not found to be altered by genetic predisposition to OSA in our MR analysis. This result is in accordance with a number of observational studies that demonstrate that individuals with OSA had the same prevalence of hypothyroidism as the general population (43, 44). Although some observational studies have reported a comparatively significant prevalence of hypothyroidism in OSA individuals (13, 45), there are some possible explanations for this discrepancy in results. First, these observational studies may often be subject to reverse causality. In addition, this discrepancy can be attributed to unmeasured confounding factors in observational research, including gender, age, and iodine nutrition status. Further research is necessary in order to illuminate pertinent discrepancies.

Bioinformatics has been a vital tool in biomedical research in recent years, particularly in the identification of potential treatment targets and the clarification of disease mechanisms. In this study, bioinformatics methods, including Venn diagrams and GO enrichment analysis, were utilized to provide insight into the potential connection between hypothyroidism and OSA. The analysis of the Venn diagrams revealed a number of DEGs that hypothyroidism and OSA have in common. This provides genetic support for the idea that hypothyroidism and OSA are strongly correlated. PPI network analysis of common DEGs identified the top 10 hub genes. NLRP3 is an inflammasome which activity is directly correlated with apnea-hypoventilation index and hypoxemia index in patients with OSA, and the damage that OSA causes to endothelial cells, neurons, the kidney, and the lung can be made worse by the activation of NLRP3 inflammasomes (46, 47). At the same time, studies show that hypothyroidism activates the inflammasome-NLRP3 pathway and thyroid hormone deficiency increases the amount of cardiac NLRP3 protein in rats (48, 49). Both NR4A1 and TREM2 are regulated by thyroid hormones and promote papillary thyroid cancer progression (50–53). Furthermore, NR4A1 is upregulated in hypothyroid juvenile mouse liver and higher sTREM2 in cerebrospinal fluid is associated with poor self-reported sleep characteristics and sleep indicators (54, 55). ATF3 is a hypoxia-associated gene biomarker for OSA and acts as a tumor suppressor in thyroid cancer (56, 57). GO enrichment analysis of the DEGs in common between hypothyroidism and OSA revealed the involvement of ERK signaling pathway, regulation of interleukin-1, and oxidative stress process. Previous studies have shown that hypothyroidism promotes increased phosphorylation of ERK1 and ERK2 and that the ERK signaling pathway is robustly activated in OSA (58, 59). The serum levels of the proinflammatory cytokine IL-1β are significantly elevated in patients with OSA (60), and high concentrations of IL-1β inhibit thyroid cell function (61). OSA results in intermittent hypoxia during sleep due to recurrent upper airway obstruction, and the resulting oxidative stress can lead not only to complications of sleep–wake rhythms, but also systemic dysfunction (62). Hypothyroidism has been shown to be associated with oxidative stress in animals and humans, thyroid hormones can modulate antioxidant levels and tissue hypothyroidism exacerbates oxidative stress (63). Thus, we consider that hypothyroidism and OSA share a common pathogenesis that may involve ERK signaling pathway, interleukin-1, and oxidative stress process. Synthesizing these bioinformatics analyses, we consider that there are complex interactions between hypothyroidism and OSA, including multiple biological pathways and mechanisms, which provided further validation of the findings of the MR analyses in the present research.

There are advantageous and limited aspects to this research. The present study is the initial effort to synergistically integrate GWAS and GEO data, using MR analysis and bioinformatics analysis to clarify the relationship of causation between hypothyroidism and OSA. To guarantee the robustness and timeliness of the findings, we used the recent large-scale GWAS data in our study. In addition, the MR analysis of the present research underwent strict quality control based on three fundamental assumptions and sensitivity analysis. There were several limitations on this study as well. Firstly, this study primarily investigates the genetic causality between hypothyroidism and OSA, while some non-genetic factors such as environmental and lifestyle factors might also have some influence. Secondly, the ability to generalize to other ethnic groups is limited due to the fact that the present study’s participants were predominantly of European descent. Furthermore, the lack of comprehensive demographic detail in the GWAS summary data prevented the conduct of in-depth subgroup analyses, including those that accounted for age and gender. As a result, subsequent research should endeavor to conduct further subgroup analyses, and where conditions permit, it could be worthwhile to conduct high-quality randomized controlled trials in an effort to derive more dependable conclusions.

## Conclusion

In conclusion, we have provided evidence that genetically predicted hypothyroidism increases the risk of OSA. Therefore, patients suffering from hypothyroidism should be intensively screened for OSA for early diagnosis and appropriate treatment. However, the effect of OSA on hypothyroidism was not found in this MR study. Further research is needed regarding the exact mechanism that contribute to the relationship of causation between hypothyroidism and OSA.

## Data Availability

The original contributions presented in the study are included in the article/Supplementary material, further inquiries can be directed to the corresponding author/s.

## Footnotes

^1^ https://gwas.mrcieu.ac.uk/

^2^ https://www.finngen.fi/en

^3^ http://www.phenoscanner.medschl.cam.ac.uk/

^4^ http://ncbi.nlm.nih.gov/geo

^5^ http://www.string-db.org/
