# Supplementary material for: Association between hypothyroidism and obstructive sleep apnea: a bidirectional Mendelian randomization study combined with the geo database
Source: Front Neurol. 2024 Dec 10;15:1420391. doi: 10.3389/fneur.2024.1420391 (PMC11666497; doi:10.3389/fneur.2024.1420391)
Supplement: Supplementary file 1 [file Data_Sheet_1.docx]

***Supplementary Material***

**Association between hypothyroidism and obstructive sleep apnea: A bidirectional Mendelian randomization study Combined with the Geo Database**

**Mingyu Zhao 1†, Xu Huang 1†, Hu Zheng1, Yuhang Cai1, Wenjia Han1, Yuanyin Wang1*, Ran Chen1***

**†These authors contributed equally to this work.**

^1^College & Hospital of Stomatology, Anhui Medical University, Key Lab. of Oral Diseases Research of Anhui Province, No.81, Meishan Road, Shushan District, Hefei City, 230032 China.

**Supplementary Figures**


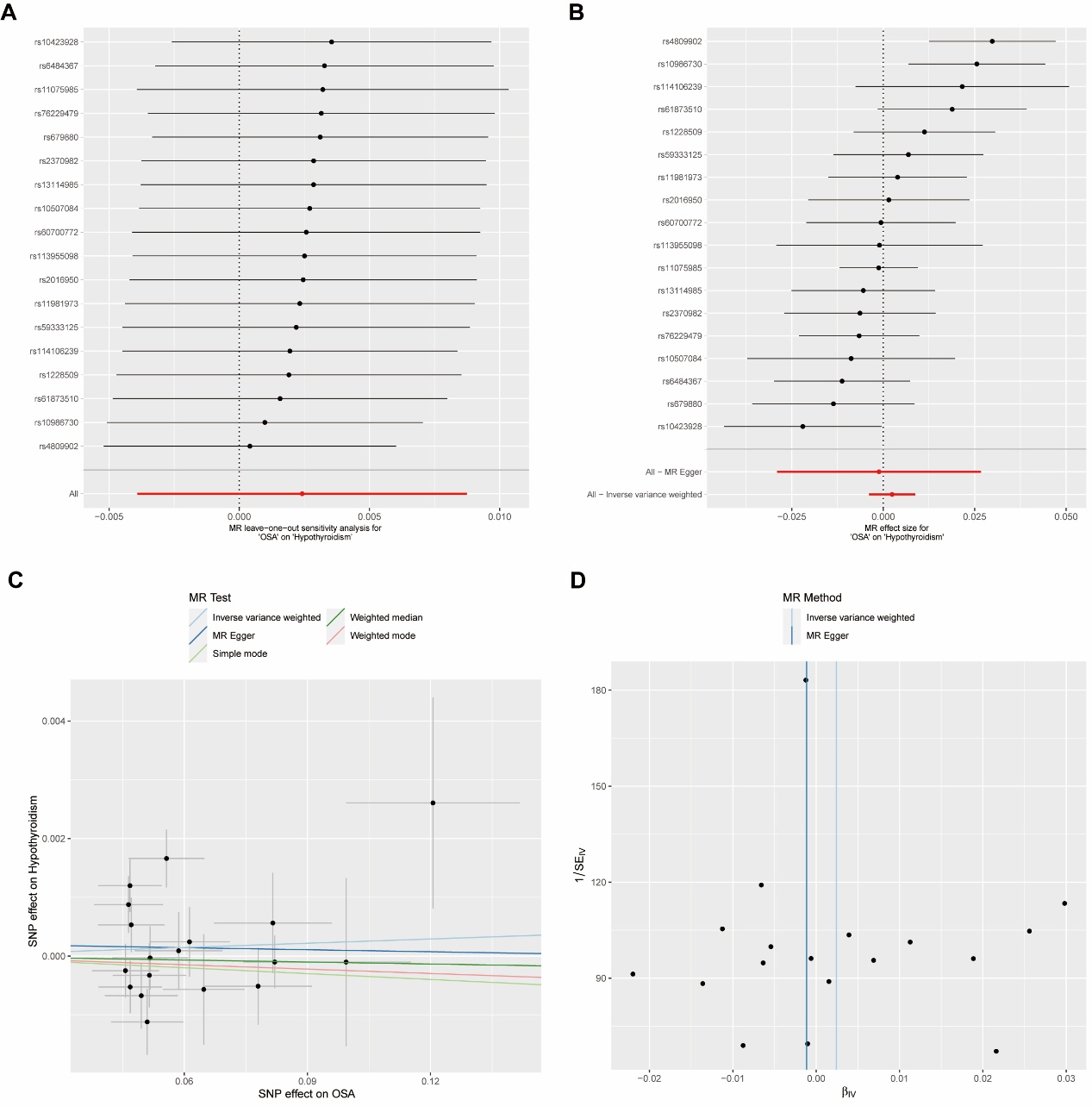


**Figure S1.** Sensitivity analysis of casual effect of OSA on hypothyroidism. (A) MR leave-one out sensitivity analysis for OSA on hypothyroidism. (B) MR effect size for OSA on hypothyroidism. (C) MR test scatterplot of five methods. (D) Funnel plot of individual SNP analyses.

**Table S1.** SNPs used as valid instrumental variables for hypothyroidism and OSA. ****

rs1921309, rs174599 and rs7754251 were removed for containing incompatible alleles or being palindromic with intermediate allele frequencies.

SNPs, single-nucleotide polymorphisms. OSA, obstructive sleep apnea. Chr, chromosome. EA, effect allele. OA, other allele. SE, standard error. EAF, effect allele frequency.

**Table S2.** SNPs used as valid instrumental variables for OSA on hypothyroidism.

rs679880 was removed for containing incompatible alleles or being palindromic with intermediate allele frequencies.

SNPs, single-nucleotide polymorphisms. OSA, obstructive sleep apnea. Chr, chromosome. EA, effect allele. OA, other allele. SE, standard error. EAF, effect allele frequency.
